# Association between sleep duration and myopia among Chinese children during the COVID-19 pandemic: A cross-sectional study

**DOI:** 10.3389/fpubh.2022.1015138

**Published:** 2023-01-09

**Authors:** Luoming Huang, Xuelan Chen, Jiajia Lin, Xianming Fan, Ting Chen, Yang Yu, Jiaxin Chen, Jianmin Hu

**Affiliations:** ^1^Department of Ophthalmology and Optometry, The School of Medical Technology and Engineering, Fujian Medical University, Fuzhou, China; ^2^Department of Ophthalmology, The Second Affiliated Hospital of Fujian Medical University, Quanzhou, China; ^3^The Research Center for Juvenile Myopia Prevention and Control of Fujian Province, Fuzhou, China; ^4^Engineering Research Center of Assistive Technology for Visual Impairment, Fujian Province University, Quanzhou, China; ^5^Eye Institute and Affiliated Xiamen Eye Center of Xiamen University, Xiamen, China; ^6^Shenzhen Eye Hospital, Shenzhen, China

**Keywords:** sleep, myopia, axial length, children, COVID-19

## Abstract

**Background:**

The studies on the association between sleep duration and myopia are limited, and the evidence is inconsistent. This study aimed to evaluate the association between sleep duration and myopia, cycloplegic spherical equivalent (SE) and axial length (AL) among Chinese children during the Corona Virus Disease 2019 (COVID-19) pandemic.

**Methods:**

The study was a cross-sectional study on Chinese children aged 6–18 years. The comprehensive ophthalmic examinations for children included cycloplegic SE, AL, and standardized questionnaires. The questionnaire included sleep duration, parental myopia, outdoor time, and continuous near work duration without breaks. Myopia was defined as SE ≤-0.50 diopters (D).

**Results:**

A total of 1,140 children were included in the analyses, with 84.7% of myopic children and 74.4% of children's daily sleep duration being more than 8 h/d. In univariate regression analysis, compared with sleep duration < 8 h/d, children with sleep duration of 8–9 and >9 h/d were less myopia (*p* < 0.01 for all), and had less myopic SE (*p* < 0.01 for all), and shorter AL (*p* < 0.01 for all). After adjusting for age, gender, parental myopia, outdoor time, and continuous near work duration without breaks, sleep duration was not associated with myopia, cycloplegic SE, and AL (*p* > 0.05 for all).

**Conclusions:**

This study showed sleep duration was related to myopia, cycloplegic SE, and AL among Chinese children during the COVID-19 pandemic-related lifestyles, but no independent association.

## 1. Introduction

Myopia, as a global public health problem, affects the vision health of children and adolescents (1). The prevalence of myopia among children in China is increasing, and the myopia rate among college students has been more than 85% (2). High myopia increases the risk of ocular diseases such as cataracts (3), glaucoma (4), myopic maculopathy (5) and retinal detachment (5). The increase in myopia has brought a substantial economic burden to society (6).

Large-scale population-based epidemiological studies have extensively investigated the risk factors for myopia. Genetic and environmental factors have been evidenced to be associated with myopia. In particular, changes in lifestyle and environment, such as short sleep duration, less outdoor activities and spending more time near work and electronic devices, are critical factors in myopia development (7–9). From the perspective of public health, changing lifestyle habits is an effective means of disease prevention, such as increasing outdoor activities can effectively reduce the incidence of myopia (10). Recently, studies have concentrated on sleep, one of the modifiable lifestyle habits. Epidemiological studies have provided preliminary evidence that sleep disorders or shorter sleep duration may influence the incidence or progression of myopia (11, 12). Jee et al. found that refractive error increased by 0.1 D for each per-hour increase in sleep duration in Korean adolescents aged 12–19 years (11). The study on Japanese children aged 10–19 indicated that children with high myopia had the poorest sleep quality and shortest sleep duration than children with emmetropia (12). In contrast, sleep duration was unrelated to myopia in studies of Chinese and Singaporean children (13–15). These inconsistent results complicate the relationship between sleep and myopia. Besides, due to the global pandemic of COVID-19, many countries have implemented social restrictions, including citywide lockdowns, home isolation, and school closures (16). These restrictions result in lifestyle changes dramatically, such as more screen time (17), less outdoor activities (18), and more sleep duration (19) among children. Compared with pre-COVID, the prevalence of myopia in children increased significantly (20). Given these, our study aimed to assess the association between sleep duration and myopia, cycloplegic spherical equivalent (SE), and axial length (AL) among Chinese children during the COVID-19 pandemic.

## 2. Methods

### 2.1. Study population

From February to December 2021, children aged 6–18 years in Fujian Province, southeastern China, were recruited to participate in this study, and the study's purpose and procedures were explained to the children and guardians. Verbal consent was obtained from each child, and written permission from the parents or guardians. The study received ethical approval from the Ethics Committee of The Second Hospital of Fujian Medical University (No. 2022244) and followed the principles of the Declaration of Helsinki. The children and their parents or guardians both agreed to the premise of using cycloplegic eye drops for dilated ophthalmic examination. Children with vision-threatening severe eye diseases, such as congenital cataracts, ocular trauma, strabismus, and amblyopia, were excluded from the study.

### 2.2. Ophthalmic examination and questionnaire

Trained ophthalmologists and optometrists conducted ophthalmic examinations of the child. Cycloplegia was induced using 3 drops of 0.5% tropicamide phenylephrine (Santen, Osaka, Japan), administered to both eyes at 5-min intervals. Pupil dilation and pupil light reflex were examined 15 min later to determine whether complete cycloplegia was achieved (pupil dilation ≥6 mm and loss of pupil light reflex). If the pupil light reflex or pupil dilation < 6 mm was still present, drops of 0.5% tropicamide phenylephrine were administered again to both eyes. Refraction was measured using autorefraction (KR-8900, Topcon, Tokyo, Japan); Axial length (AL) was measured using AL-Scan (Nidek, Gamagori, Japan). All measurements were performed 3 times, and the mean value was calculated for each eye. If the difference between any two readings in one eye was ≥0.5 D, the refraction of that eye was remeasured. Spherical equivalent (SE) was calculated as the refraction of the sphere power plus half the cylinder power. Myopia was defined as SE ≤-0.50 D.

The online questionnaire was completed by the child or guardian (the child who could not complete the questionnaire independently) and asked to describe the behavior in the past month. The questionnaire included demographic information, parental myopia, the daily duration of sleep, the daily duration spent on outdoor activities, and continuous near work duration without breaks. The questions were designed according to the Chinese National Health Commission recommendation (21): sleep duration <8 h/d, 8–9 h/d, > 9 h/d. Outdoor activities time options were categorized as <2 h/d and ≥2 h/d. How long was the duration of a single continuous near work without breaks, options were categorized as < 30 min and ≥ 30 min.

### 2.3. Statistical analysis

Because of highly correlated data of cycloplegic SE and AL for the right and left eyes (Pearson correlation coefficient (r) =0.84 for SE and 0.92 for AL), only the right eye data was included in the analyses. Continuous non-parametric data were expressed as median and interquartile range and analyzed using the Mann-Whitney U test or Kruskal-Wallis test; categorical variables were analyzed using the Chi-square or Fisher exact test. Myopia, cycloplegic SE, and AL were used as dependent variables (outcomes), and sleep duration categorical variables were used as independent variables (exposures) to assess the association using univariate logistic or linear regression models; multivariate logistic or linear regression models were conducted to adjust for confounders such as age, gender, parental myopia, outdoor time, and continuous near work duration without breaks. Statistical software SPSS 24.0 (Statistical Product Service Solutions, version 24, Chicago, IL, USA) was used for analysis. Statistical significance was assumed at *p* < 0.05 with two-sided.

## 3. Results

A total of 1,206 participants were enrolled in the study. Excluding the subjects with missing data and those who did not meet the inclusion criteria, 1,140 children were available for statistical analysis (Table 1). Sleep duration was significantly associated with myopia (*p* < 0.01). Parental myopia, outdoor time, and continuous near work duration without breaks were not associated with myopia (*p* > 0.05 for all).

**Table 1:** Characteristics of children stratified by myopia status.

**Variable**	**Total**	**Non-myopia**	**Myopia**	***p***
Age (years), *n* = 1,140	12 (9, 14)	9 (7, 11)	12 (9,14)	<0.01^†^
SE (D), *n* = 1,140	−1.88 (−3.13, −0.88)	0.13 (−0.25, 0.50)	−2.19 (−3.50, −1.25)	<0.01^†^
AL (mm), *n* = 957	24.0 (23.69, 25.06)	23.0 (22.68, 23.69)	25.0 (23.97, 25.25)	<0.01^†^
Gender				0.09^*^
Boys	552 (48.4%)	74 (42.5%)	478 (49.5%)	
Girls	588 (51.6%)	100 (57.5%)	488 (50.5%)	
Sleep duration, h/d				<0.01^*^
<8	292 (25.6%)	28 (16.1%)	264 (27.3%)	
8–9	548 (48.1%)	93 (53.4%)	455 (47.1%)	
>9	300 (26.3%)	53(30.5%)	247 (25.6%)	
Parental myopia				0.27^*^
None	439 (38.5%)	70 (40.2%)	369 (38.2%)	
One	410 (36.0%)	68 (39.1%)	342 (35.4%)	
Both	291 (25.5%)	36 (20.7%)	255 (26.4%)	
Time outdoor, h/d				0.26^*^
<2 h/d	123 (10.8%)	23 (13.2%)	100 (10.4%)	
≥2 h/d	1,017 (89.2%)	151 (86.8%)	866 (89.6%)	
Continuous near work duration without breaks, min				0.49^*^
<30	133 (11.7%)	23 (13.2%)	110 (11.4%)	
≥30	1,007 (88.3%)	151 (86.8%)	856 (88.6%)	

† Analyzed using Mann-Whitney U-test.

* Analyzed using Chi-square test.

Data are presented as medians and quartiles (p25, p75) or as n (%).

SE, spherical equivalent; D, diopters; AL, axial length.

Table 2 shows the SE and AL stratified by sleep duration and gender. Myopia was associated with sleep duration (*p* < 0.01). Myopic SE and AL significantly decreased with increasing sleep duration for all children and girls (*p* < 0.01 for all). Myopia was not associated with sleep duration among boys (*p* = 0.57).

**Table 2:** The spherical equivalent and axial length stratified by sleep duration and gender.

**Variable**	***n***	**Myopia**	p^*^	***n***	**SE, D**	***p* ^†^**	***n***	**AL, mm**	***p* ^†^**
Total	1,140			1,140			957		
Sleep duration, h/d			<0.01			<0.01			<0.01
<8	292	264 (90.4%)			−2.50 (−4.13, −13.80)			24.71 (24.04, 25.55)	
8–9	548	455 (83.0%)			−1.63 (−2.88, −0.75)			24.30 (23.58, 24.95)	
>9	300	247 (82.3%)			−1.69 (−2.88, −0.75)			24.25 (23.65, 24.87)	
Boys	552			552			460		
Sleep duration, h/d			0.57			<0.01			<0.01
<8	130	116 (89.2%)		130	−2.56 (−4.13, −1.38)		114	25.14 (24.57, 25.84)	
8–9	268	229 (85.4%)		268	−1.75 (−3.13, −0.88)		229	24.61 (24.04, 25.37)	
>9	154	133 (86.4%)		154	−1.88 (−3.00, −0.88)		117	24.64 (24.00, 25.12)	
Girls	588			588			497		
Sleep duration, h/d			<0.01			<0.01			<0.01
<8	162	148 (91.4%)		162	−2.38 (−4.00, −1.38)		142	24.44 (23.77, 25.04)	
8–9	280	226 (80.7%)		280	−1.50 (−2.75, −0.63)		237	23.87 (23.28, 24.59)	
>9	146	114 (78.1%)		146	−1.44 (−2.75, −0.50)		118	23.85 (23.31, 24.50)	

* Analyzed using Fisher exact test.

† Analyzed using Kruskal-Wallis test.

Data are presented as medians and quartiles (p25, p75) or as n (%).

SE, spherical equivalent; D, diopters; AL, axial length.

In univariate logistic regression model analysis, compared with sleep duration <8 h/d, children with sleep duration of 8–9 and >9 h/d had less myopia (*p* < 0.01 for all) in all children (Table 3). In univariate linear regression model analysis, compared with sleep duration < 8h/d, children with sleep duration of 8–9 and >9 h/d had less myopic SE (*p* < 0.01 for all) and shorter AL (*p* < 0.01 for all) in all children. However, after adjusting for potential confounders such as gender, age, parental myopia, outdoor time, and continuous near work duration without breaks, sleep duration was not significantly associated with myopia, cycloplegic SE, and AL (*p* > 0.05 for all).

**Table 3:** Univariable and multivariable analysis of sleep duration in participants.

	**Myopia**				**SE, D**				**AL, mm**			
**Variable**	**Univariable model**		**Multivariable model**		**Univariable model**		**Multivariable model**		**Univariable model**		**Multivariable model**	
	**OR** **[95%CI]**	***p*** ^†^	**OR** **[95%CI]**	***p*** ^†^	**Coefficient** β **[95%CI]**	**p** ^*^	**Coefficient** β **[95%CI]**	**p** ^*^	**Coefficient** β **[95%CI]**	**p** ^*^	**Coefficient** β **[95%CI]**	**p** ^*^
**Total**												
Sleep duration, h/d											
<8	Ref		Ref		Ref		Ref		Ref		Ref	
8–9	0.52 [0.33, 0.81]	<0.01	1.29 [0.78, 2.16]	0.34	0.78 [0.52, 1.04]	<0.01	−0.01 [−0.28, 0.25]	0.92	−0.44 [−0.61, −0.28]	<0.01	0.06 [−0.10, 0.21]	0.47
>9	0.50 [0.30, 0.81]	<0.01	1.57 [0.88, 2.79]	0.13	0.79 [0.50, 1.08]	<0.01	−0.23 [−0.54, 0.08]	0.15	−0.49 [−0.68, −0.30]	<0.01	0.13 [−0.05, 0.31]	0.16
**Boys**												
Sleep duration, h/d											
<8	Ref		Ref		Ref		Ref		Ref		Ref	
8–9	0.71 [0.37, 1.36]	0.30	1.76 [0.83, 3.74]	0.14	0.67 [0.27, 1.07]	<0.01	−0.16 [−0.55, 0.23]	0.42	−0.42 [−0.65, −0.18]	<0.01	0.11 [−0.11, 0.33]	0.34
>9	0.77 [0.37, 1.57]	0.47	2.60 [1.10, 6.13]	0.03	0.73 [0.29, 1.17]	<0.01	−0.39 [−0.84, 0.06]	0.09	−0.50 [−0.77, −0.24]	<0.01	0.16 [−0.10, 0.42]	0.23
**Girls**												
Sleep duration, h/d											
<8	Ref		Ref		Ref		Ref		Ref		Ref	
8–9	0.40 [0.21, 0.74]	<0.01	0.96 [0.47, 1.97]	0.91	0.90 [0.56, 1.24]	<0.01	0.15 [−0.21, 0.5]	0.43	−0.53 [−0.73, −0.32]	<0.01	0.11 [−0.21, 0.22]	0.96
>9	0.34 [0.17, 0.66]	<0.01	1.02 [0.46, 2.26]	0.96	0.87 [0.48, 1.27]	<0.01	−0.08 [−0.51, 0.34]	0.69	−0.54 [−0.78, −0.30]	<0.01	0.13 [−0.15, 0.36]	0.41

Ref indicates reference.

† Analyzed using logistic regression analysis.

* Analyzed using linear regression analysis.

Multivariable model adjusted for age, gender, parental myopia, outdoor time and continuous near work duration without breaks.

SE, spherical equivalent; D, diopters; AL, axial length; OR, odds ratio; CI, confidence interval.

## 4. Discussion

The present study showed sleep duration was related to myopia, cycloplegic SE, and AL among Chinese children during the COVID-19 pandemic-related lifestyles, but no independent association.

In this study, boys had a more myopic SE (Z = −2.18, *p* = 0.029) and longer AL (Z = −10.24, *p* < 0.01) than girls, both of which were statistically different. The data was similar to that of Shanghai and Wuhan children in China (22, 23). Studies assessing the association between sleep duration and myopia have not been widely conducted, and findings vary. Our results show that sleep duration was no independent relation to myopia, cycloplegic SE, and AL among Chinese children. Similar to Singapore (GUSTO) study showed no association between sleep duration and myopia, SE, or AL among 376 3-year-old children (24). Recently, Li et al. also found no independent correlation between sleep duration and myopia, SE, or AL in the same GUSTO study in 572 9-year-old Singaporean children (15). Moreover, the study by Liu et al. of 4,982 Chinese children aged 6–9 years revealed that late bedtime but not sleep duration related to 2-year myopia onset (25). Additionally, the 4-year follow-up study of 1,187 children aged 5–9 years indicated that sleep duration and bedtime were not significantly associated with myopia progression and axial elongation after adjusting for potential confounders (13). In contrast, a study of 3,625 Korean adolescents aged 12–19 years showed that sleep duration was negatively associated with myopia and showed a dose-response pattern (11). Similarly, Ayaki et al. (12) found in 278 Japanese children aged 10–19 years that children with high myopia had the shortest sleep duration compared to other groups (*p* < 0.01). In a study of Chinese children aged 6–18 years, Gong et al. (26) found a progressively lower risk of myopia in children who slept about 8 and 7 h/d or less compared to those who slept 9 h/d or more (OR,2.12; 95%CI, 1.94~2.31; OR, 3.37; 95%CI, 3.07~3.70, respectively). We speculate that this may be due to non-cycloplegic refraction and different ages leading to inconsistent results. Non-cycloplegic refraction may overestimate the prevalence of myopia in children (27), leading to refractive category errors. In our study, the extensive age range is also an important confounding factor that cannot be ignored. The prevalence of myopia increases with age, and sleep duration decreases gradually, which is related to the vast educational pressure on children. Additionally, our study was conducted during COVID-19 pandemic and children's activities were limited. Their lifestyles changed dramatically (28, 29), such as increased screen time (17) and decreased time outdoors (18), and their sleep would also be affected (19). People's lifestyle is also different from usual in a specific living environment, and children's myopic shifts are changed. Several studies reported higher myopia incidence (by around 2.0–2.6 fold) among Chinese children compared to pre-COVID in the period during COVID-19 (20, 30–32). The narrow sleep duration classification (74.4% children more than 8 h/d) and higher myopic prevalence (84.7% myopic children) may partially explain the inconsistent results of our study.

At present, the mechanism between sleep and myopia is not precise. A potential mechanism explanation was that the disorder of circadian rhythm might be related to the onset and development of myopia. When the eyes develop to high myopia, the pathological changes of intrinsically photosensitive retinal ganglion cells (ipRGCs) may be caused by the tissue changes of the retina due to axial elongation. Animal model studies have demonstrated that myopia progression may increase the risk of retinal stretch and damage, damaging the ipRGCs in the inner retina (33, 34). And ipRGCs are closely related to light reception and circadian rhythm. In patients with blindness and cataracts, reduced transmittance and photoreception in the eyes lead to disturbances in circadian rhythm and sleep disorders (35). There is a distinct circadian rhythm in children's eyes, with intraocular pressure, AL, choroidal thickness, and retinal thickness all showing particular rhythmic changes (36). Dopamine expression levels have significant circadian rhythmicity and are regulated by the rhythm clock and melatonin, which have an essential role in myopia development (37). Stone et al. suggested that retinal rhythm and clock genes may underlie refractive control (38). Bmal1 is an important molecule that regulates rhythm, and it has been reported that retina-specific Bmal1 knockout mice of different ages exhibit a myopic compared to wild-type mice. The phenotype suggests that rhythm abnormalities may be associated with myopia (39). Chakraborty et al. found that myopia was associated with delayed melatonin circadian rhythm phasing in young adults and lower melatonin output (40).

The strengths of this study included the large sample size and the use of cycloplegic SE and AL, thus minimizing errors in estimating SE and myopia categories. The online questionnaire provided a convenient way to address the questionnaire, minimizing the entry errors of paper questionnaires during the transfer to digital data. Additionally, we provided data on children's sleep duration and myopia in specific lifestyles during the COVID-19 pandemic. However, there were several limitations should be mentioned. First, sleep duration was collected from questionnaires, and the results may be subject to recall bias resulting in reduced accuracy. Although the differences in sleep duration obtained using the questionnaire and polysomnography were minimal (41). Questionnaires to collect sleep data may be a suitable method in studies with large samples. Second, sleep data did not include bedtime, wake time, and sleep quality. However, prior studies could not provide sufficient evidence to support the association of sleep quality and bedtime with myopia (15, 42, 43). Third, the study did not include screen time in children. More screen time might lead to decrease sleep duration. Finally, the present study was cross-sectional and could not clarify the causal relationship between sleep duration and myopia. And Our study conducted during the COVID-19 pandemic with specific lifestyles might not be representative to clarify these associations, and more studies on unspecific lifestyles are needed to confirm.

## 5. Conclusions

In conclusion, this study showed no independent association between sleep duration and myopia, cycloplegic SE and AL among Chinese children during the COVID-19 pandemic-related lifestyles. Cohort studies with precise sleep data to confirm the relationship between sleep and myopia should be conducted in unspecific lifestyles.

## Data availability statement

The raw data supporting the conclusions of this article will be made available by the authors, without undue reservation.

## Ethics statement

The studies involving human participants were reviewed and approved by the study obtained ethical approval from the Ethics Committee of The Second Hospital of Fujian Medical University (No. 2022244) and followed the principles of the Declaration of Helsinki. Written informed consent to participate in this study was provided by the participants' legal guardian/next of kin.

## Author contributions

LH and JL designed the study. LH, XC, and JL drafted the initial manuscript. JL and XC collected the data. LH, JL, and TC analyzed the data. LH, XC, JL, XF, TC, YY, JC, and JH reviewed and edited the manuscript. JH acts as a guarantor of the study. All authors reviewed and approved the final version of the manuscript.
